# Injectable anti-inflammatory, antioxidant supramolecular nanofiber hydrogel for peripheral nerve injury repair and neuropathic pain relief

**DOI:** 10.1016/j.mtbio.2026.102780

**Published:** 2026-01-07

**Authors:** Anqi Liu, Kui Sheng, Huihui Li, XinYang Zhao, Xiaojun Zhang, Haitao Su, Tsring Samdrup, Ye Zhang, Xianwen Hu, LiJun Weng, YaGuang Wang

**Affiliations:** a Department of Pain, The Second Affiliated Hospital of Anhui Medical University, 678 Furong Road, Hefei, 230601, PR China; b Department of Anesthesiology, The Second Affiliated Hospital of Anhui Medical University, 678 Furong Road, Hefei, 230601, PR China; c Key Laboratory of Anesthesiology and Perioperative Medicine of Anhui Higher Education Institutes, Anhui Medical University, 678 Furong Road, Hefei, 230601, PR China; d Department of Anesthesiology, Fuyang People's Hospital Affiliated to Anhui Medical University, 501 Sanqing Road, Fuyang, Anhui, PR China; e Department of Anesthesiology, People's Hospital of Shannan City, Tibet Autonomous Region, PR China

**Keywords:** Peripheral nerve injury, Supermolecular hydrogel, Curcumin, Anti-inflammatory, Antioxidant, Neuropathic pain

## Abstract

Patients with peripheral nerve injury (PNI) often experience neuropathic pain (NP), which is difficult to treat effectively due to ongoing inflammation and oxidative stress that impede nerve repair. Traditional anti-inflammatory antioxidants are limited by short half-lives and significant side effects. This study introduces a supramolecular hydrogel formed by combining anti-inflammatory betamethasone phosphate (Betp) with calcium chloride to create betamethasone phosphate hydrogel (Betp@Gel). The antioxidant curcumin (Cur) was incorporated into this gel to form Cur/Betp@Gel, which can be injected directly at the injury site for sustained Cur release. Cur/Betp@Gel demonstrates superior sustained-release capabilities and therapeutic effectiveness compared to Betp@Gel or Cur alone. It enhances pain relief, supports functional recovery after nerve damage, and promotes nerve repair and regeneration. This is achieved by Betp@Gel's anti-inflammatory effects, which inhibit TNF-α, IL-1β, and IL-6, combined with Cur's continuous slow release, which scavenges reactive oxygen species (ROS). Additionally, Cur/Betp@Gel mitigates PNI-induced spinal inflammation by reducing GFAP and Iba-1 expression in the spinal cord. Overall, in-situ injection of Cur/Betp@Gel is a promising strategy for aiding nerve repair and alleviating neuropathic pain.

## Graphical abstract

Anti-inflammatory and antioxidant drugs can effectively alleviate the inflammation and oxidative stress responses caused by peripheral nerve injury. Therefore, in this study, we designed a supramolecular hydrogel with betamethasone phosphate (Betp) as the hydrogelator and loaded the antioxidant curcumin (Cur) to assemble an injectable Cur/Betp@Gel for in situ injection. Cur/Betp@Gel demonstrates superior sustained-release capabilities and therapeutic effectiveness compared to Betp@Gel or Cur alone. It enhances pain relief, supports functional recovery after nerve damage, and promotes nerve repair and regeneration. This is achieved by Betp@Gel's anti-inflammatory effects, which inhibit TNF-α, IL-1β, and IL-6, combined with Cur's continuous slow release, which scavenges reactive oxygen species (ROS). Additionally, Cur/Betp@Gel mitigates PNI-induced spinal inflammation by reducing GFAP and Iba-1 expression in the spinal cord. Overall, in-situ injection of Cur/Betp@Gel is a promising strategy for aiding nerve repair and alleviating neuropathic pain.

**Figure:**
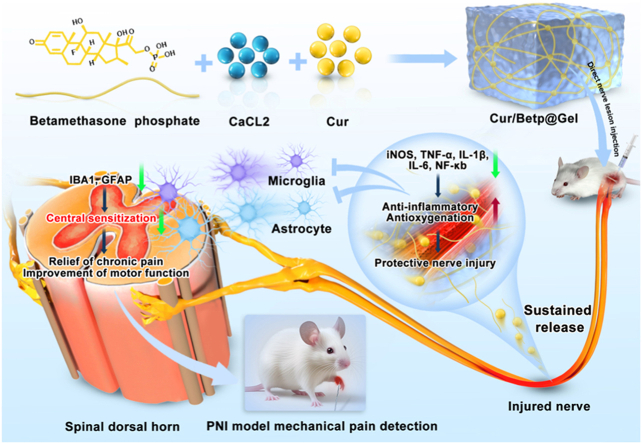

\

## 1 Introduction

Peripheral nerve injury (PNI) frequently results from accidental trauma, acute compression, or medical procedures, leading to nerve dysfunction [1]. Following PNI, patients may develop neuropathic pain (NP), characterized by impaired sensation, spontaneous pain, abnormal pain responses (allodynia, hyperalgesia), and increased sensitivity [[2], [3], [4]].

The inflammatory response following PNI plays a crucial role in NP development [5]. Chronic NP resulting from PNI may arise due to persistent inflammation stemming from an autoimmune response to nerve damage [6]. Both pro-inflammatory and anti-inflammatory responses are activated at the site of injury post-PNI; however, the pro-inflammatory response predominates, leading to peripheral and central sensitization [7]. Notably, pro-inflammatory cytokines (e.g., TNF-α, IL-1β, IL-6) and chemokines produced by M1 macrophages at the injury site can directly influence sensory neuron excitability [8]. Excessive inflammation can cause tissue damage and impair regeneration [9], increasing the risk of chronic pain [10,11].

Furthermore, the surge in reactive oxygen species (ROS) following PNI not only intensifies nerve damage but also exacerbates pain by modulating neuronal excitability and inflammatory responses [12]. NADPH oxidase 2 (Nox2)-derived ROS play a significant role in activating spinal microglia and subsequent pain hypersensitivity [13]. Injured neurons, reactive glial cells, and immune cells generate ROS and reactive nitrogen species (RNS), exacerbating nerve damage, neuroinflammation, and peripheral sensitization, thereby extending neuroinflammation and facilitating chronic NP persistence [13].

Basic studies on treating chronic pain after PNI involve antioxidants [14,15], and anti-inflammatory [16], which can repair injured nerves and reduce chronic pain occurrence. Current therapeutic strategies for PNI-related pain often involve anticonvulsants, antidepressants, and opioids. However, these treatments do not promote injury recovery and frequently cause severe side effects [17]. Relevant studies suggest that effective anti-inflammatory and antioxidant treatments can significantly enhance the proliferation of various neuronal subtypes and effectively repair injured nerves [18]. Injecting steroid hormones around injured peripheral nerves is widely used clinically and has demonstrated efficacy in treating peripheral neuropathies, such as chronic inflammatory demyelinating polyradiculoneuropathy and post-herpetic neuralgia [19,20].

Betamethasone phosphate (Betp), a commonly used anti-inflammatory steroid, is preferred for treating peripheral neuralgia. As a glucocorticoid, it suppresses the NF-κB signaling pathway and pro-inflammatory cytokines, reducing inflammation [21] Perineural steroid injection achieves high local drug concentrations near inflamed nerve roots, effectively alleviating pain and improving functional outcomes [22]. However, PNI triggers inflammation lasting up to 4 weeks [23], while Betp has a short in vivo half-life and rapidly loses activity at the administration site due to diffusion and enzymatic degradation [24]. Maintaining effective local drug levels requires repeated injections, increasing the risk of further nerve injury. Therefore, developing a method for localized, long-retention, sustained drug release is urgently needed. Betamethasone's short half-life necessitates repeated injections to maintain therapeutic concentrations [25]. Clinically, steroid injections often require repetition every 2–3 weeks, leading to undesirable side effects such as injection site inflammation, pituitary-adrenal axis suppression, hyperadrenocorticism, Cushing's syndrome, and osteoporosis. Repeated injections also heighten the risk of nerve damage. Consequently, there is a pressing clinical need for a biocompatible system that reduces injection frequency and cumulative steroid dose, significantly advancing nerve repair and pain alleviation after PNI.

Curcumin (Cur), containing phenol and beta-diketone groups, functions as an antioxidant by inhibiting free radicals. Its potent antioxidant properties are well-documented in studies on various chronic diseases [26]. Cur inhibits inflammatory cell proliferation and activity by blocking pro-inflammatory pathways (NF-κB, MAPK, iNOS) via its antioxidant effects. Specifically, Cur demonstrated significant analgesic effects in neuropathic pain models induced by chemotherapy [27], diabetes [28], and PNI [15], as well as in postoperative pain. However, Cur's poor water solubility compromises its clinical application, resulting in low absorption, limited distribution to target tissues, and reduced bioavailability [29]. Although various drug delivery systems (e.g., hemoglobin [30], alginate [31]) improve its bioavailability, challenges remain regarding Cur's stability and duration of action.

Amphiphilic supramolecular hydrogels have shown exceptional efficacy as drug carriers. Their specific binding properties restrict unrestricted drug molecule movement, enhancing stability and extending drug half-life. They are widely used in controlled drug release research for applications such as immunotherapy [32], MR Imaging [33], antibacterial therapy [34], tissue regeneration [35], and tumor therapy [36]. These studies suggest supramolecular hydrogels can effectively address drug off-target issues and poor local retention in injured nerves. However, their ability to continuously regulate inflammatory responses, counteract oxidative stress, improve the neuroinflammatory microenvironment, and inhibit acute and chronic pain has not been reported.

**Scheme 1:**
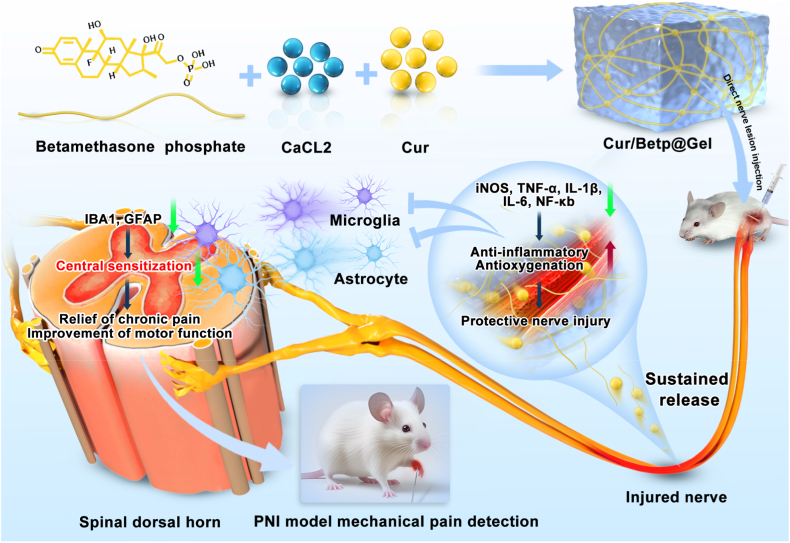
Schematic diagram of Cur/Betp@Gel injection for peripheral nerve injury (PNI) repair. The Figure above illustrates the molecular structure and composition of Betp, as well as the structure and components of Cur/Betp@Gel. The lower Figure demonstrates the effects after local injection of Cur/Betp@Gel around the injured peripheral nerve in a PNI model: reduced inflammatory response, repaired injured nerves, alleviated neuropathic pain, and improved motor function.

To address this, we designed a supramolecular hydrogel using betamethasone phosphate as the hydrogelator. Mixing Betp with a calcium chloride (CaCl_2_) aqueous solution leverages coordination between Betp's phosphate groups and Ca^2+^ ions, intermolecular hydrogen bonding between hydroxyl groups, and hydrophobic interactions between rigid steroid nuclei, rapidly forming betamethasone phosphate hydrogel (Betp@Gel) [37]. This anti-inflammatory steroid-based nanofiber hydrogel serves a dual purpose: inhibiting inflammation and facilitating curcumin loading to form Cur/Betp@Gel. We hypothesized that injecting Cur/Betp@Gel around injured nerves would enable slow, continuous release of Betp and curcumin, continuously regulating nerve regeneration and repair while inhibiting acute and chronic pain through anti-inflammatory and antioxidant activities (Scheme 1).

## 2 Materials and methods

### 2.1 Preparation of composite hydrogels

The construction of Betp@Gel was achieved by uniformly mixing an aqueous Betp solution with an equal volume of CaCl_2_ solution, resulting in immediate gelation. For the preparation of the drug-loaded system Cur/Betp@Gel, a Cur dissolution system was first prepared: dimethyl sulfoxide (10 % v/v) was used as the primary solvent, followed by sequential addition of polyethylene glycol 300 (40 % v/v), Tween 20 (5 % w/v), and physiological saline (45 % v/v) to construct a composite solvent system. The Cur solution was thoroughly integrated with the Betp (12.5 mM) aqueous solution via ultrasonic dispersion combined with vortex oscillation. Finally, CaCl_2_ solution was introduced to initiate the gelation reaction, and the thermocycling process yielded the drug-loaded hydrogel with a final Cur concentration of 0.433 mg/mL.

### 2.2 Material characterization and rheological analysis

Microstructural characterization was performed using transmission electron microscopy (TEM, Talos L120C G2, Thermo Fisher Scientific™). Sample pretreatment included: droplet deposition of the gel dispersion onto a copper grid, adsorption for 120 s, rinsing with ultrapure water to remove free substances, and subsequent drying. Morphological observations were conducted under an accelerating voltage of 120 kV.

The mechanical properties of the hydrogels were evaluated using a rotational rheometer system (MCR 302e, Anton Paar, Austria). This included dynamic frequency sweep (0.1–10 Hz, γ = 1 %) to determine the frequency dependence of the storage modulus (G') and loss modulus (G''), strain sweep (0.01–1 %, f = 1 Hz) to investigate the linear viscoelastic region, and temperature response and shear-thinning tests (f = 1 Hz, γ = 1 %). All test samples had a uniform volume of 500 μL and underwent pre-equilibration prior to experimentation.

### 2.3 Establishment of nerve injury model and intervention protocol

Experiments utilized male Sprague Dawley rats (SPF grade, aged 8–10 weeks, body weight 200–250 g) under experimental animal use license LLSC20242241. Randomized groups included: sham operation group (Sham, 200 μL phosphate buffer solution), peripheral nerve injury group (PNI), blank hydrogel group (Betp@Gel), free drug group (Cur) (0.433 mg/mL), and drug-loaded hydrogel group (Cur/Betp@Gel), with each group administered 200 μL. After anesthesia with sodium pentobarbital (30 mg/kg, i. p.), a right hindlimb incision was made to expose the sciatic nerve trunk. A standard hemostatic clamp was applied with 30 g pressure for 2 min to establish a chronic compression model. Postoperative wound disinfection and layered suturing were strictly performed. Animals were transferred to cages for postoperative management after recovery.

### 2.4 In vitro Cur and release from Cur/Betp@Gel

Cur/Betp@Gel was transferred into a 2 mL microcentrifuge tube. The sample was kept at 37 °C during incubation by slowly shaking on a shaker. 500 μL supernatant was extracted at predetermined intervals (1, 2, 4, 8, and 16 days) for subsequent analysis and supplemented with equal volumes of fresh phosphate buffer solution (PBS) (pH 7.4). After extraction, the supernatant sample is kept at −20 °C for analysis. The amount of Cur discharged into the supernatant was quantified via absorbance at 425 nm using a microplate reader. At the same time, high performance liquid chromatography (HPLC) was used to quantify the amount of Betp excreted from the supernatant at each specified time interval, making it possible to assess its cumulative release over the specified duration.

Dynamic dialysis evaluated the sustained-release profile of Cur/Betp@Gel. First, the mass loss method was used to evaluate the in vitro degradation kinetics of Cur/Betp@Gel. The experimental group was set up as follows: equal-volume hydrogel samples were immersed in PBS at pH 8.0, 7.4, and 6.8. The dynamic degradation experiments were then conducted in a constant-temperature oscillating incubator at 37 °C and 100 rpm. Samples were taken out at the preset time nodes (1, 2, 4, 8, and 16 days), the supernatant was removed by a precision pipetting system, and the surface liquid film was adsorbated by aseptic filter paper. The remaining hydrogel wet weight (Wt) was determined immediately by microbalance. Mass retention rate (%) is calculated by the formula (Wt/W_0_) × 100 %, where W_0_ is the initial wet weight.

### 2.5 In vivo imaging of Betp@Gel retention in PNI rats

In order to determine the retention and distribution of Betp@Gel in the body after injection, Cy7.5 fluorescent dye was encapsulated into Betp@Gel to construct the Cy7.5/Betp@Gel tracing system. Cy7.5-labeled Betp@Gel was injected into the site of sciatic nerve injury. Pure Cy7.5 solution served as the control. The fluorescence signal of the anesthetized rats was measured and the mean value of the fluorescence signal was analyzed statistically by Tannon imaging system on the 1st, 3rd and 5th day post injection.

### 2.6 Biosafety evaluation of the drug-loaded system

Cell viability assay (CCK-8), F11 cells were seeded in 96-well plates. After 24 h, the original culture medium was aspirated. Cells were then treated with extracts prepared by immersing different concentrations of Betp@Gel or equivalent concentrations of Betp and CaCl_2_ mixtures (0, 3.125, 6.25, 12.5, 25 mM) in DMEM high glucose medium supplemented with 10 % fetal bovine serum (FBS) and 0.1 % penicillin-streptomycin for 24 h at 37 °C. Cells incubated in complete medium throughout the entire process served as the control group. All groups were incubated at 37 °C in a humidified atmosphere with 5 % CO_2_ for an additional 24 or 48 h. Subsequently, cells were washed three times with sterile PBS. Following washing, 110 μL of pre-prepared CCK-8 working solution was added to each well, and the plates were incubated for 3 h under the same conditions (37 °C, 5 % CO_2_). After incubation, 100 μL of the CCK-8 working solution from each well was transferred to a new 96-well plate. The absorbance (OD) at 450 nm was measured using an enzyme-linked immunosorbent assay (ELISA) plate reader. Cell viability was calculated based on the recorded OD values.

The systemic toxicity of Cur/Betp@Gel was evaluated through serum biochemical indicators and histopathological methods. On the 5th day after PNI surgery, rat serum was collected and quantitatively analyzed using commercial kits (ALT: S03030, Rayto; AST: S03040, Rayto; γ-GT: S03031, Rayto). On the 28th day, the rats were euthanized to obtain heart, spleen, lung, kidney and adrenal gland tissues. After fixation in 4 % formaldehyde, paraffin sections were prepared and stained with hematoxylin-eosin (HE). The morphological changes of parenchymal cells and interstitial inflammatory infiltration were graded and evaluated under an optical microscope.

### 2.7 In vitro validation of cur antioxidant activity

An oxidative stress model was established using the F11 cell line (rat dorsal root ganglion-derived, ECACC origin). Cells were cultured in high-glucose DMEM (10 % fetal bovine serum, 1 % penicillin-streptomycin) at 37 °C under 5 % CO_2_ (Servicebio Technology. Cat. No. G4511). Experimental groups (Cur, Betp@Gel, Cur/Betp@Gel) were co-treated with 200 μM H_2_O_2_ for 24 h, washed thrice with DPBS, and stained with 1 μM DCFH-DA probe under light protection for 30 min. ROS signals were captured using fluorescence microscopy (Olympus IX73, Japan), and mean fluorescence intensity was quantified via ImageJ (ImageJ, Maryland, USA). Statistical analysis employed one-way ANOVA.

### 2.8 In vitro anti-inflammatory effects of Betp@Gel and Cur/Betp@Gel

RAW264.7 macrophages (Shanghai Fuhong Biotech) were cultured in high-glucose DMEM (Servicebio G4511) supplemented with 10 % fetal bovine serum and 1 % penicillin-streptomycin. Groups included negative control (DMSO), LPS stimulation, and treatment groups (Cur, Betp@Gel, Cur/Betp@Gel). After 24-h pretreatment, all groups except the negative control were stimulated with 100 ng/mL LPS (Solarbio Co. Ltd, Cat. No. L8880) for 8 h. Culture supernatants were centrifuged (1000×*g*, 5 min) to remove debris. ELISA kits quantified IL-6 (Thermo Fisher), IL-1β (Thermo Fisher), and TNF-α (Thermo Fisher) concentrations, with triplicate experiments ensuring reliability.

### 2.9 von Frey test for mechanical allodynia

Mechanical pain thresholds were assessed using von Frey filaments (IITC2393; IITC Life Science, USA). Rats were acclimated to the testing apparatus for ≥30 min. A series of von Frey filaments (2.0 g) were applied to the plantar skin. Rapid paw withdrawal or persistent licking was recorded as a positive response. Each animal underwent five trials (15-min intervals). Withdrawal thresholds were defined as the pressure eliciting ≥3 withdrawal responses.

### 2.10 Hargreaves test for thermal hyperalgesia

Thermal withdrawal latency was measured using a Hargreaves apparatus (IITC390G; IITC Life Science, USA). Rats were stabilized in a temperature-controlled observation cage for 30 min. A calibrated heat source (intensity 30) was focused on the affected plantar epidermis. Latency from stimulus onset to withdrawal was recorded automatically, with a 25-s cutoff to prevent tissue damage. Three measurements per rat (15-min intervals) were averaged.

### 2.11 Gait analysis

A motorized treadmill (RWD, MSI DigiGait) assessed post-injury motor function at 20 cm/s. High-speed cameras recorded the gait cycles of each group of rats. MSI DigiGait software calculated the sciatic functional index (SFI): SFI = 100 × [(experimental side parameter − contralateral parameter)/contralateral parameter]. SFI approaching 0 indicates normal function; 100 % denotes complete dysfunction. Double-blind data collection was performed by two independent observers over 28 days.

### 2.12 Open field behavioral test

Male C57 mice (8–12 weeks old, 22–28 g) were used similarly to the PNI rat model. They were randomly assigned to treatment groups receiving 20 μL of Betp@Gel, Cur, or Cur/Betp@Gel. During surgery, mice were anesthetized with pentobarbital sodium (30 mg/kg, intraperitoneally) to expose the sciatic nerve. A hemostatic forceps applied pressure for 2 min to create a chronic compression model. Post-surgery, wounds were disinfected and sutured in layers. After recovery, the mice were moved to cages for postoperative care.

Mice were acclimated to the open field for 30–60 min. The arena was cleaned with ethanol and dried to eliminate odor interference. Animals were placed facing away from the experimenter in the center. SMART 3.0 software recorded and analyzed 10-min locomotion.

### 2.13 Gastrocnemius histological analysis

On the 28th day following PNI, all rats in each experimental group were euthanized. The gastrocnemius muscle tissues from the affected side were excised, fixed in 4 % paraformaldehyde, and subsequently embedded in paraffin to produce sections with a thickness of 10 μm. These sections were stained with hematoxylin and eosin and examined microscopically. The mean diameters of the muscle fibers were assessed by analyzing four randomly selected images.

### 2.14 Peripheral nerve histological evaluation

Nerve specimens (injury center ±5 mm) of PNI rats underwent two processing protocols. Paraffin sections: Dehydrated in graded ethanol, transversely sectioned (10 μm), and stained with HE for nerve fiber structure or Toluidine Blue (TB) staining for myelin integrity. Whole-slide imaging used an Olympus VS200 system.

Frozen sections: Dehydrated in 30 % sucrose, OCT-embedded, and sectioned (10 μm). Immunohistochemical staining included: 3 % H_2_O_2_ (peroxidase inactivation), 3 % BSA (blocking), primary antibodies (S100β: GB11336, Servicebio, 1:100; GAP43: AF0240, Beyotime, 1:200) at 4 °C for 16 h, HRP-conjugated goat anti-rabbit IgG (A0208, Beyotime, 1:50) for 50 min at room temperature, and DAB visualization with hematoxylin counterstaining. Imaging used a microscope (Vert A1, Zeiss, Germany).

### 2.15 Immunofluorescence of injured nerves

On the 7th day after PNI, the nerve tissues (injured center ±5 mm) harvested from each group of rats were fixed in 4 % paraformaldehyde, then dehydrated in 30 % sucrose, and embedded using OCT. Sagittal frozen sections (10 μm) were permeabilized with 0.3 % Triton X-100 (3 × 5 min), incubated with primary antibodies (iNOS: AF0199, Affinity, 1:100; TNF-α: GB11188, Servicebio, 1:200; Arg-1: 93668, CST 1:200) at 4 °C for 18 h, followed by Alexa Fluor 488-conjugated donkey anti-rabbit IgG (A-21206, Invitrogen, 1:500) or goat anti-rabbit Cy3 (gb21203, 1:500) for 2 h in darkness, and DAPI nuclear staining. Images were captured via confocal microscopy (Vert A1, Zeiss, Germany). ImageJ quantified positive cell ratios.

### 2.16 Spinal glial activation

L4–L6 spinal cord segments harvested from each group of rats at day 28 after PNI were fixed, dehydrated, and transversely sectioned (10 μm). Immunofluorescence staining included: 0.3 % Triton X-100 permeabilization, 5 % donkey serum blocking, primary antibodies (GFAP: AF0156, Beyotime,1:100; Iba-1:AF7143, Beyotime,1:50) at 4 °C for 16 h, and Alexa Fluor 594-conjugated donkey anti-mouse IgG (A-21203, Invitrogen, 1:500). Fluorescence images (exposure 500 ms, gain 2.0) were acquired via fluorescence microscopy (Vert A1, Zeiss, Germany) and analyzed with ImageJ.

### 2.17 ELISA

ELISA quantified inflammatory cytokines in nerve tissues harvested from each group of rats at 7days after PNI. A 10 mm sciatic nerve segment (injury center) was homogenized in ice-cold RIPA buffer (1 % protease inhibitors) for 60 min. Lysates were centrifuged (12,000×*g*, 15 min, 4 °C), and total protein was quantified via BCA assay. TNF-α (Thermo Fisher), IL-6 (Thermo Fisher), and IL-1β (Thermo Fisher) levels were measured using commercial kits (Invitrogen) and normalized to pg/mg total protein. Similarly, 28 days post-PNI, rats from each group underwent ELISA analysis to assess the expression of IBA1(MLBio) and GFAP (MLBio), following the resection of the L4-6 spinal cord segments as previously described.

### 2.18 Western blot analysis

To assess changes in protein expression related to acute neuroinflammation and long-term nerve repair after injury, this study employed Western Blot analysis to quantify total and phosphorylated NF-κB, S100, and Gap43. The procedure involved anesthetizing rats, perfusing them with a cold PBS, excising a 1 cm section of the injured nerve, and extracting total protein using a lysate. Protein concentration was determined via the BCA method, samples were diluted to a uniform concentration, and heated at 100 °C for 10 min. Proteins (20 μg) were separated using 10 % SDS-PAGE and transferred to a polydifluoroethylene membrane. After the membrane transition is completed, all membranes are sealed for 20 min with a protein-free rapid closure solution at room temperature. Then add the specific resistance and incubate it in a 4 °C environment overnight. The antibodies used and their dilution ratio include: NF-κB (CST, 1:1000), P- NF-κB (CST, 1:1000), Gap43 (CST, 1:1000), S100β (Abcam, 1:1000), GAPDH (Proteintech, 1:1000). Then, use Tris buffer salt solution containing 0.24 % Tween-20 to clean the film three times for 10 min each time. After washing, it was incubated at room temperature for 1 h with horseradish peroxidase-labeled rabbit or mouse antibodies respectively. After cleaning again, use the chemiluminescent substrate kit for development. Finally, the image is collected through the gel imaging system, and the NIH ImageJ software is used to analyze the grayscale value of the target strip.

### 2.19 Electron microscopy

On the 28th day after PNI, 3–5 mm long segments of the sciatic nerve tissue were removed from the distal end of the injured area of the rat. Then they were placed in a 2.5 % glutaraldehyde solution and fixed at 4 °C for pre-fixation. After fixing, rinse the sample four times with PBS for 1 h each. Immerse the tissue in 1 % yttrium acid solution for 2 h, then wash three times with deionized water for 10 min each. Stain with 2 % uranium acetate for 2 h, followed by gradient dehydration with ethanol and acetone. Embed in epoxy resin, cure, and slice into 70 nm sections. Stain with lead citrate and dry. Observe with a Thermo Scientific Talos L120C G2 transmission electron microscope. For analysis, select four images and use ImageJ to measure myelin sheath thickness of all nerve fibers.

### 2.20 Statistical analysis

Data are presented as mean ± SEM. Normality was verified via Shapiro-Wilk test. Group differences were analyzed using: unpaired two-tailed Student’s t-test, one-way ANOVA with Tukey’s post hoc test, or two-way repeated-measures ANOVA with Dunnett’s correction for time-series data. All analyses were performed in GraphPad Prism 9.0 (GraphPad Software Inc., San Diego,CA), with significance denoted as ∗p < 0.05, ∗∗p < 0.01, ∗∗∗p < 0.001, ∗∗∗∗p < 0.0001.

## 3 Results

### 3.1 Morphological features of Betp@Gel and Cur/Betp@Gel

**Fig. 1:**
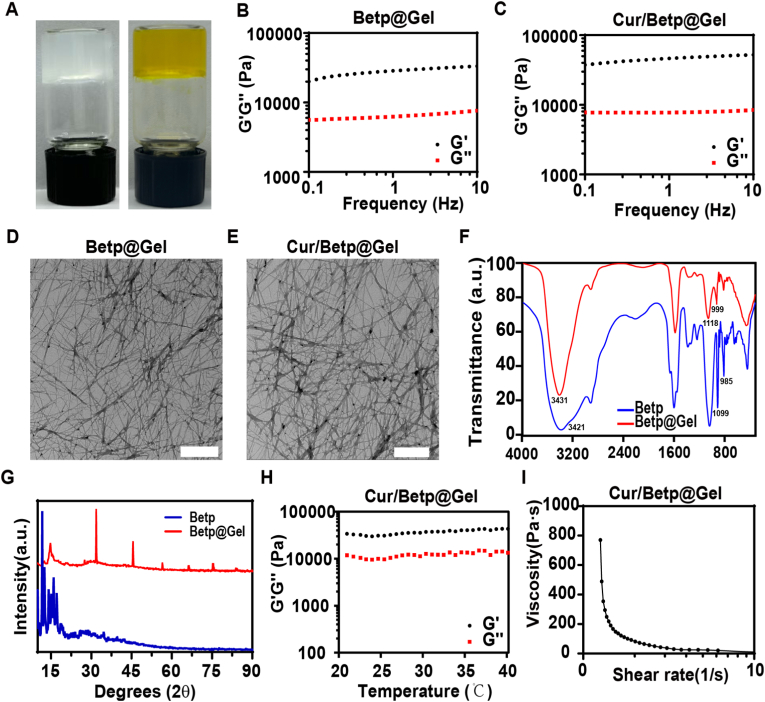
The characterization of Cur/Betp@Gel. (A) Photographs showing Betp@Gel (left) and Cur/Betp@Gel (right). Storage modulus (G′) and loss modulus (G″) of Betp@Gel (B) and Cur/Betp@Gel (C) at different frequencies with a strain rate of 1 %. Transmission electron microscopy (TEM) images of nanofibers: Betp@Gel (D) and Cur/Betp@Gel (E). Scale bar: 500 nm. (F) FTIR spectra of Betp powder and Bet@Gel. (G) XRD patterns of Betp powder and Betp@Gel. a. u., arbitrary unit. (H) Stability evaluation of Cur/Betp@Gel at varying temperatures under a test frequency of 1 Hz and 1 % strain. (I) Shear rate versus viscosity changes of Cur/Betp@Gel.

To understand the molecular properties of the Betp, we performed a detailed molecular analysis using ^1^H NMR (Fig. S1), and ^13^C NMR (Fig. S2). First, Betp and Cacl2 were mixed at a 1:1molar ratio. It can be observed that as the concentration increased, the mixture gradually formed a solid hydrogel. Firstly, Betp and Cacl2 were mixed at a 1:1molar ratio. It can be observed that as the concentration increased, until the mixture concentration reached 12.5 mM, a solid hydrogel sufficient for self-support could be formed (Fig. S3). Fig. 1A illustrates that the combination of 12.5 mM Betp with an equivalent concentration of CaCl2 does not impede hydrogel formation, even when the encapsulated Cur concentration is 0.433 mg/mL (retained in the inverted bottle). Rheological analysis demonstrated that the storage modulus (G') of both hydrogels consistently exceeded the loss modulus (G'') across frequency (0.1–10 Hz, γ = 1 %) (Fig. 1B and C) and strain (0.01–1%, f = 1 Hz) sweeps (Fig. S4A and S4B), confirming their elastic network properties. As shown in Fig. 1D and E, the TEM images showed that both Betp@Gel and Cur/Betp@Gel exhibited a three-dimensional reticular nanofiber structure. The dense nanofiber networks within the Cur/Betp@Gel, measuring 18.2 ± 4.7 nm in diameter and 142.2 ± 45.72 nm pore size. The above indicated that the loading of Cur/Betp@Gel on Cur did not change the gel mechanical properties.

To investigate how Betp@Gel forms, FTIR and XRD were used to characterize both Betp and the Betp@Gel. According to the FTIR data, the bands specific to the phosphate group moved from 985 to 1099 cm^−1^ to higher wave numbers of 999 and 1118 cm^−1^, indicating the formation of coordination bonds with Ca^2+^ (Fig. 1F). XRD patterns contrasted sharply: Betp powder exhibited crystalline peaks (5–45°), while Betp@Gel showed amorphous features (broad peaks at 7.0°, 15.0°) attributed to a BetP-Ca^2+^ complex, evidencing phosphate-Ca^2+^ coordination (Fig. 1G).

Temperature-dependent studies (20–40 °C) showed stable G' values for Cur/Betp@Gel (Fig. 1H). Shear-thinning behavior under increasing shear rates (Fig. 1I) confirmed injectability and in situ retention.

### 3.2 Biocompatibility of Cur/Betp@Gel

The potential toxicity of biomaterials is the main obstacle to their biomedical applications. Therefore, it is necessary to conduct biocompatibility tests to evaluate the toxicity of Cur/Betp@Gel. First, we evaluated cytocompatibility in the Cur/Betp@Gel cell line. As shown in Fig. S6, the results of the cell viability test indicated that Cur/Betp@Gel did not affect the viability of F11 cells, suggesting good cytocompatibility in vitro.

A systematic analysis of blood biochemistry and histopathology of visceral tissues was conducted to evaluate the potential short-term and long-term toxicity of Cur/Betp@Gel following perisciatic injection in rats. Liver function indicators in the Cur/Betp@Gel group were all within the normal range. This indicates that Cur/Betp@Gel is safe and reliable in the short term after local injection into the body (Fig. S7A, B and 7C). Histological examination revealed no significant histopathological abnormalities or lesions in the major organs (heart, liver, spleen, lungs, kidneys, adrenal glands) of the rats sacrificed 28 days after hydrogel injection confirmed that no tissue toxicity was found in all groups (Fig. S8, Supporting Information).

### 3.3 Retention effect of Cur/Betp@Gel in the PNI model

**Fig. 2:**
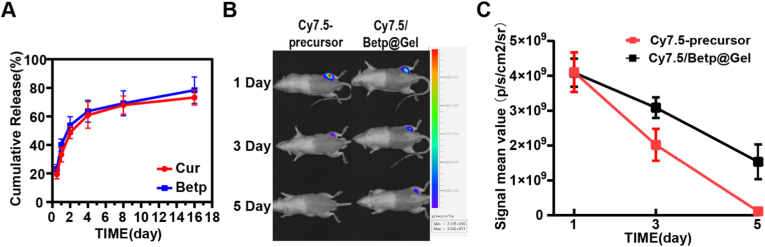
(A) In vitro release rate of Cur. (B) Retention of Cur/Betp@Gel injected with Cy7.5 at 1, 3 and 5 days post-injection, detected via Tanon IVIS analysis. (C) Fluorescence signal analysis of in vivo retention after 1, 3, 5 days of simple injection of Cy7.5 and Cy7.5/Betp@Gel.

In vitro, as shown in Fig. 2A, under the action of simulated shear force combined with hydrolysis, the Cur released by Cur/Betp@Gel reached 67.95 ± 6.38 % on the 8th day, and the Betp released reached 69.21 ± 8.78 % on the 8th day. This finding offers substantial evidence for the sustained drug release characteristics of Cur/Betp@Gel in vivo.

In vitro simulation experiments, the following experimental conditions were used to construct different physiological microenvironments. The PBS buffer at pH 7.4 and 37 °C mimics the normal physiological environment, while PBS at pH 6.8 and pH 8.0 simulate acidic and alkaline microenvironments, respectively. Shaking the shaker replicates in vivo shear forces. As shown in Fig. S5, during the observation period of 28 days, the degradation curve of Cur/Betp@Gel showed that the material system could meet the basic stability requirements of implant materials in vivo.

Through in vivo imaging of animals, the retention of pure Cy7.5 solution injected around the sciatic nerve and Cy7.5/Betp@Gel loaded with Cy7.5 in the body was observed (Fig. 2B and C). The signal from pure Cy7.5 solution gradually disappeared within 3 days, whereas the signal from Cy7.5/Betp@Gel persisted for at least 5 days, confirming the sustained-release and strong retention properties of Cur/Betp@Gel.

### 3.4 Antioxidant effects and anti-inflammatory mechanisms of cur and Cur/Betp@Gel in vitro

**Fig. 3:**
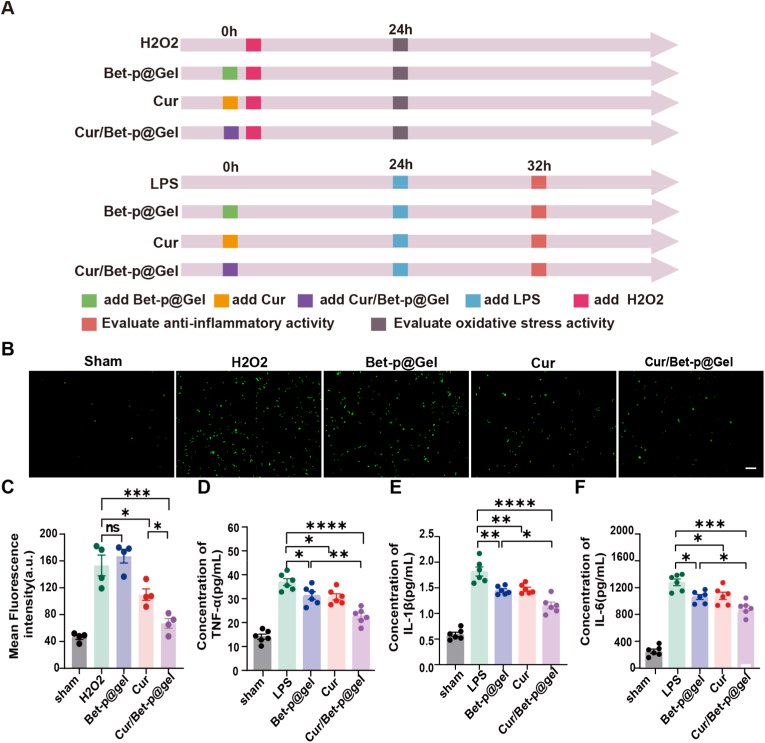
Anti-inflammatory and antioxidant effects of Cur/Betp@Gel in vitro. (A) LPS-induced macrophage inflammation model and H_2_O_2_-induced oxidative stress model in spinal dorsal root neuron cells. (B) Representative fluorescent images of oxidative stress after ROS probe intervention. Scale bar: 50 μm. (C) Statistical analysis of average fluorescence intensity post-oxidative stress across groups. (D–F) ELISA-based statistical analysis of inflammatory markers (TNF-α, IL-1β, IL-6) in macrophage supernatants (n = 6). Data are presented as the means ± SEM. ∗P < 0.05, ∗∗P < 0.01, ∗∗∗P < 0.001.

Furthermore, we established an in vitro oxidative stress model in spinal dorsal root ganglion neurons using H2O2 induction (Fig. 3A). Our results demonstrated that compared to the Sham group, the H2O2-only group exhibited significantly stronger fluorescence from the ROS probe, indicating more severe oxidative stress. Both the Cur group and Cur/Betp@Gel group effectively prevented oxidative stress induction, with Cur/Betp@Gel showing superior efficacy (P < 0.05). In contrast, no significant difference was observed between the Betp@Gel group and H2O2 group. These findings suggest that Cur may serve as the key active component in this system for free radical scavenging (Fig. 3B and C).

In addition, we established an in vitro inflammatory model using cell experiments (Fig. 3A). ELISA results measuring the inflammatory markers TNF-α, IL-1β, and IL-6 in macrophage supernatants (Fig. 3D–F) demonstrated that compared to the LPS group, all treatment groups effectively reduced these inflammatory markers. Notably, The Cur/Betp@Gel group exhibited a more significant anti-inflammatory effect compared to the other two treatment groups (P < 0.05).

### 3.5 Antioxidant effects and anti-inflammatory mechanisms of cur, Betp-Gel, and Cur/Betp@Gel validated in vivo in the acute stage of nerve injury

**Fig. 4:**
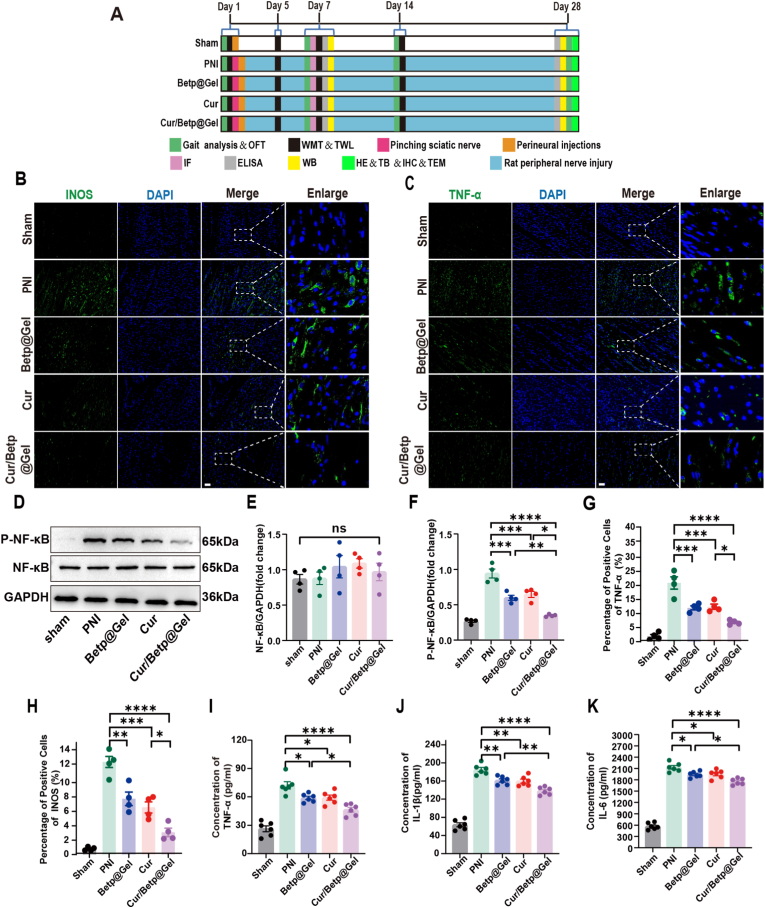
Validation of the antioxidant and anti-inflammatory mmechanisms of Cur/Betp@Gel in vivo. (A) Experimental animal grouping and timeline for molecular assays and behavioral observations. (B, C) Representative immunofluorescence staining images of iNOS and TNF-α in injured sciatic nerves of the rats 7 days post-PNI. Scale bar: 50 μm. (D) Representative Western blot images of p-NF-κB, NF-κB and GAPDH protein expression in nerves of the rats 7 days post-PNI injury. (E, F) Quantitatively analyze the expression level of p-NF-κB, NF-κB were analyzed using ImageJ software (n = 4). (G, H) Statistical analysis of iNOS and TNF-α-positive areas (n = 6). (I–K) ELISA-based statistical analysis of TNF-α, IL-1β, and IL-6 levels in sciatic nerve tissues (n = 6). Data are presented as the means ± SEM. ∗P < 0.05, ∗∗P < 0.01, ∗∗∗P < 0.001, ∗∗∗∗P < 0.0001.

All in vivo experiments followed the flowchart in Fig. 4A, with rats randomly assigned to five groups: Sham, PNI, Betp@Gel, Cur, and Cur/Betp@Gel. On the 7th day following PNI (Fig. 4B–H), immunostaining for inducible iNOS was conducted on the injured sciatic nerves of the rats. We observed that the PNI group exhibited more prominent positive regions, indicating more severe oxidative stress. All treatment groups effectively reduced these positive regions, with both the Cur group and Cur/Betp@Gel group showing more significant alleviation of oxidative stress. Notably, the Cur/Betp@Gel group exhibited the best antioxidant effect (P < 0.0001).

TNF-α immunostaining of injured nerves confirmed severe inflammation in the PNI group (Fig. 4C–G), while treatment groups exhibited reduced positivity, with Cur/Betp@Gel showing the strongest anti-inflammatory effects (P < 0.0001). The western blot analysis of injured nerve sites showed that the expression of NF-κB in each group was not inhibited. Compared with the PNI group, the p-Nf-κb expression was significantly decreased in the Cur/Betp@Gel group and Betp@Gel group, especially in the Cur/Betp@Gel group (p < 0.0001). This suggests that the Cur/Betp@Gel group showed the potential to suppress the inflammatory response during the acute phase of nerve injury (Fig. 4D–F).

ELISA analysis of TNF-α, IL-1β, and IL-6 levels in 10 mm sciatic nerve segments showed that PNI induced significant cytokine production, which was effectively reduced by all treatments, The Cur/Betp@Gel group exhibited a more significant anti-inflammatory effect compared to the other two treatment groups (P < 0.05) (Fig. 4I–K). Arg-1 immunostaining of the injured sciatic nerve (Fig. S9A and B) revealed distinct expression patterns across groups. Compared to the PNI group, all treatment groups significantly increased positive regions. Notably, the Cur/Betp@Gel group showed the most pronounced enhancement in Arg-1 expression (P < 0.0001). This marked upregulation indicates that Cur/Betp@Gel is particularly effective in promoting the M2-like pro-repair phenotype, which is crucial for nerve regeneration.

### 3.6 Cur, Betp@Gel, and Cur/Betp@Gel alleviate PNI-induced pain and improve motor function

**Fig. 5:**
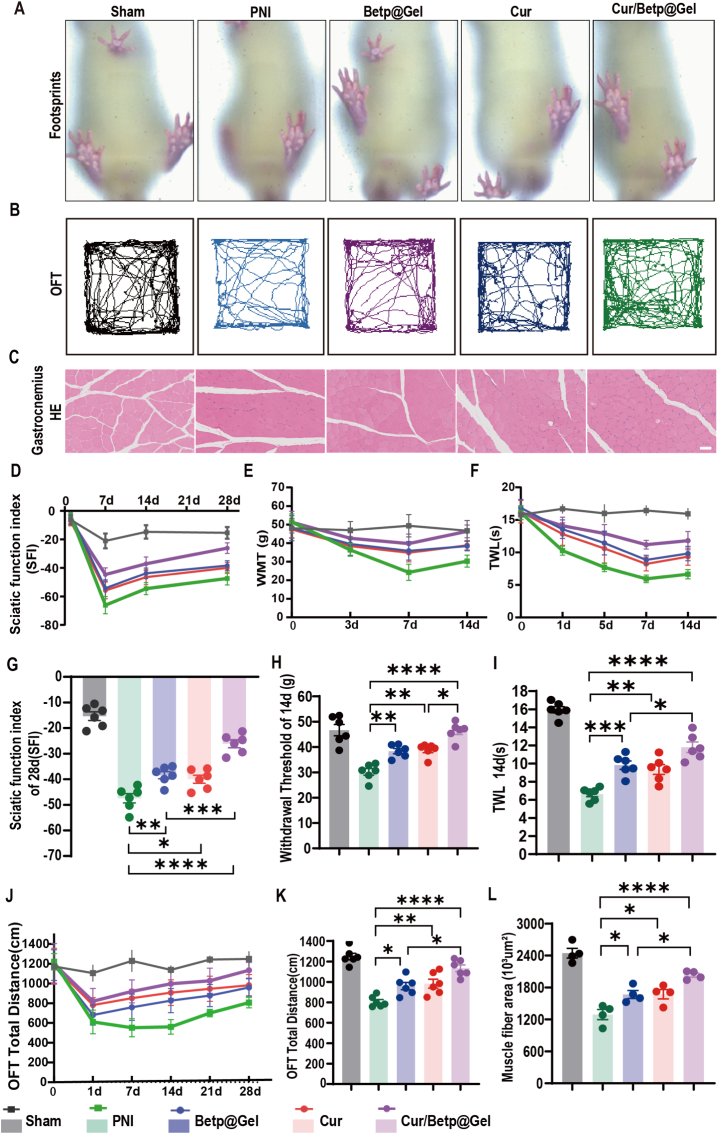
Cur/Betp@Gel alleviates inflammation and improves motor function. (A) Footprint images of left (normal) and right (injured) paws 28 days post-PNI. (B) Representative trajectory maps of mice in open-field tests at 28 days post-PNI. (C) HE-stained cross-sectional images of gastrocnemius muscles. Scale bar: 50 μm. (D) Changes in sciatic functional index (SFI) pre- and post-surgery across groups, with statistical analysis at 28 days post-PNI (G) (n = 6). (E) Changes in mechanical pain threshold (WMT) pre- and post-PNI, with statistical analysis at 14 days (H) (n = 6). (F) Changes in thermal pain latency (TWL) pre- and post-surgery, with statistical analysis at 14 days post-PNI (I) (n = 6). (J) Changes in total travel distance in open-field tests pre- and post-PNI, with statistical analysis at 28 days (K) (n = 6). (L) Statistical analysis of gastrocnemius muscle cross-sectional area (n = 4). ∗P < 0.05, ∗∗P < 0.01, ∗∗∗P < 0.001, ∗∗∗∗P < 0.0001.

Mechanical and thermal pain threshold assessments showed no pre-operative differences among groups of PNI rats. The other groups generally reached their lowest pain threshold around 1 week after surgery. Compared among the groups, the treatment groups all alleviated pain to some extent. At postoperative day 14, the Cur/Betp@Gel group showed more significant pain relief than the other two treatment groups (P < 0.05) (Fig. 5E–H). Similarly, based on the changes in thermal pain threshold, it was observed that a continuous decrease in thermal pain threshold occurred within the first week after surgery. All treatment groups effectively alleviated the development of thermal hypersensitivity. Compared to the PNI group, all treatment groups significantly reduced thermal hypersensitivity, with the Cur/Betp@Gel group demonstrating a more pronounced advantage over the other two treatment groups at 14 days after PNI (P < 0.05) (Fig. 5F–I). In addition to sensory function improvement, motor function was also assessed through walking performance (Fig. 5A) and sciatic functional index (SFI) analysis (Fig. 5D–G), revealed markedly reduced SFI values in all post-operative groups compared to Sham. However, SFI significantly improved in treatment groups versus PNI, with Cur/Betp@Gel showing the most pronounced effect. At postoperative day 28, the Cur/Betp@Gel group showed the most significant improvement compared to the other two treatment groups (P < 0.001). Open field testing (Fig. 5B) demonstrated that the PNI group had the shortest total walking distance in 10 min, while all treatment groups exhibited improved mobility, Furthermore, at day 28, the Cur/Betp@Gel group exhibited better therapeutic effects than the other treatment groups (P < 0.05) (Fig. 5J and K). Consequently, Cur, Betp-Gel, and Cur/Betp@Gel demonstrated a reduction in both pain and motor dysfunction, with Cur/Betp@Gel exhibiting the most pronounced therapeutic efficacy.

### 3.7 Cur, Betp@Gel, and Cur/Betp@Gel prevent muscle atrophy and promote nerve repair/regeneration

**Fig. 6:**
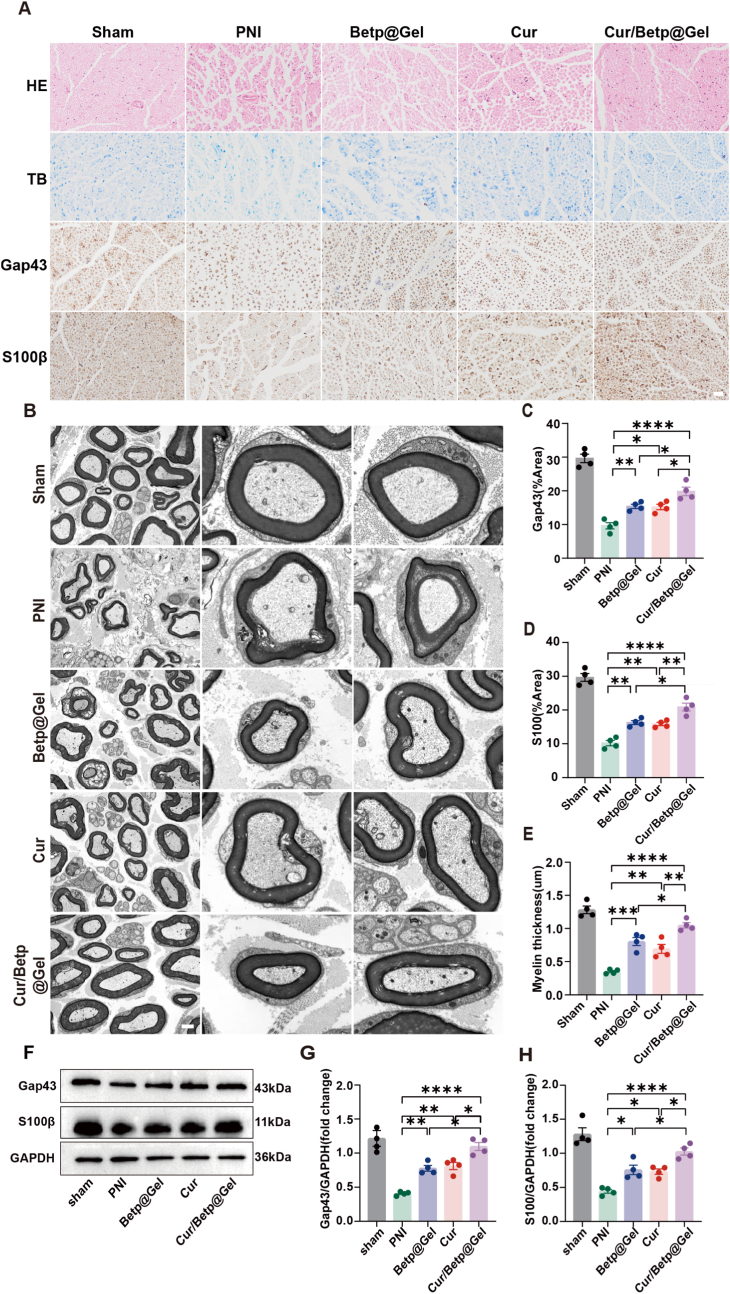
Histological analysis of regenerated sciatic nerves 28 days post-PNI in rats. (A) Representative cross-sectional images: HE staining (scale bar: 20 μm), TB staining (scale bar: 20 μm), Gap43 immunohistochemistry (scale bar: 20 μm), S100β immunohistochemistry (scale bar: 20 μm). (B) TEM image representation and local magnification of myelin regeneration (scale: 2 μm). (C) Quantification of average Gap43-positive area (%) in the mid-region of regenerated nerves (n = 4). (D) Quantification of average S100β-positive area in the mid-region of regenerated tissues (n = 4). (E) Statistical analysis of myelin thickness (n = 4). (F) Representative Western blot images of total Gap43, S100β and GAPDH protein expression injured nerves. (G, H) Quantitatively analyze the expression level of S100β and Gap43 were analyzed using ImageJ software (n = 4). Data are presented as the means ± SEM. ∗P < 0.05, ∗∗P < 0.01, ∗∗∗P < 0.001, ∗∗∗∗P < 0.0001.

Histological examination of the gastrocnemius muscle on the 28th day post- PNI (Fig. 5C–L) indicated that muscle fibers within the PNI cohort exhibited disorganization and a reduction in cross-sectional area. Conversely, all treatment groups demonstrated improvements in these parameters, with the Cur/Betp@Gel treatment exhibiting the most pronounced effect. HE and TB staining of injured sciatic nerves revealed more compact and orderly nerve bundles in the Betp@Gel, Cur, and Cur/Betp@Gel groups compared to the PNI group, with the Cur/Betp@Gel group showing the most substantial improvement (Fig. 6A). PNI induced structural disarray and shrinkage of axons and myelin sheaths. Statistical analyses revealed that the expression levels of S100β and GAP43 in Schwann cells were elevated in all treatment groups. Importantly, the Cur/Betp@Gel group exhibited significantly higher expression levels compared to the other treatment groups. (Fig. 6C and D). At the same time, the western blot analysis of the injured nerve site was further carried out. The results showed that compared with the PNI group, the expression of GAP43 and S100β of Schwan cells in each treatment group was increased, among which the Cur/Betp@Gel group performed better (P < 0.05) (Fig. 6F–H). In order to deeply explore the promoting effect of Cur/Betp@Gel on myelin regeneration, we further used a TEM to observe the ultra-microstructure of the sciatic nerve, and quantitatively evaluated the thickness of the myelin sheath based on high-resolution microscopy images (Fig. 6B–E). Statistical analysis shows that the thickness of the myelin sheath in each treatment group increased significantly compared with that in the PNI group. It is worth noting that the Cur/Betp@Gel group was particularly effective in promoting myelin thickening, and there were also statistical differences compared with other groups (p < 0.01).

### 3.8 Cur, Betp@Gel, and Cur/Betp@Gel alleviate spinal inflammatory responses induced by PNI

**Fig. 7:**
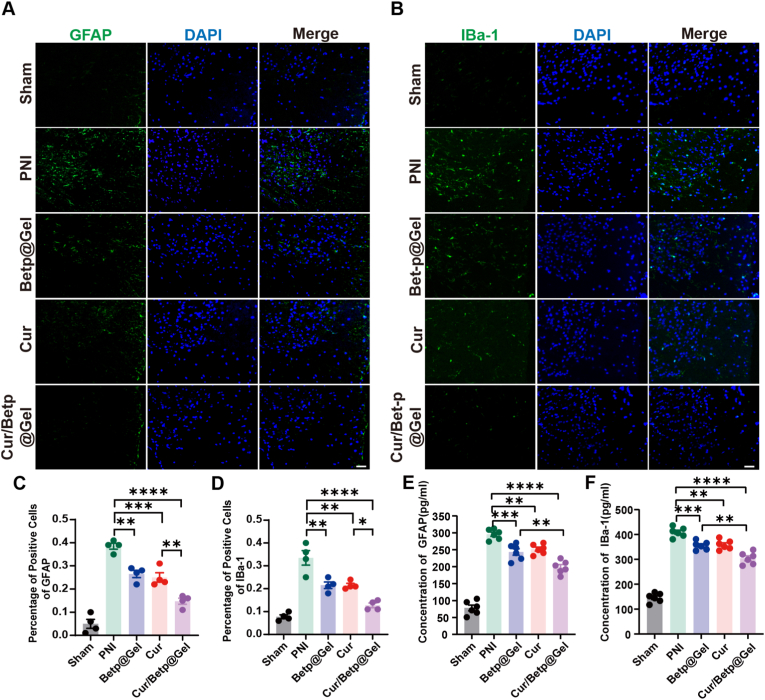
Cur/Betp@Gel suppresses PNI-induced spinal pro-inflammatory effects. (A, B) Representative immunofluorescence staining images of GFAP and Iba-1 in the L4–L6 spinal dorsal horn 28 days post-PNI in rats. Scale bar: 2 μm. (C, D) Statistical analysis of GFAP and Iba-1-positive areas in the spinal dorsal horn across groups (n = 4). (E, F) ELISA -based statistical analysis of GFAP and IBA1 levels in L4–L6 Spinal cord (n = 6). Data are presented as the means ± SEM. ∗P < 0.05, ∗∗P < 0.01, ∗∗∗P < 0.001, ∗∗∗∗P < 0.0001.

The immunofluorescence analysis of GFAP, an astrocyte marker, and Iba-1, a microglial marker, was conducted on the dorsal horn of the L4-L6 spinal cord segments in rats from each experimental group on the 28th day post-PNI (Fig. 7A and B). The results demonstrated that the expression levels of these markers were significantly elevated in the PNI group compared to the Sham group, suggesting an activation of neuroinflammatory processes. All treatments reduced GFAP and Iba-1 positivity, with Cur/Betp@Gel group exhibiting the strongest suppression of spinal pro-inflammatory responses linked to neuropathic pain (P < 0.0001) (Fig. 7C and D). Furthermore, the expression of GFAP and IBA1 were quantitatively evaluated using ELISA in spinal cord samples corresponding spinal cord segments. Consistent with the immunofluorescence, the expression levels of GFAP and IBA1 were significantly decreased in all treatment groups compared to the PNI group. Notably, the Cur/Betp@Gel group demonstrated the most pronounced reduction (P < 0.0001) (Fig. 7E and F).

## 4 Discussion

The pathological process following PNI involves inflammatory responses and oxidative stress. Traditional therapies are often limited by low drug delivery efficiency and single-target effects. Here, we constructed a Cur-loaded Betp@Gel composite hydrogel system (Cur/Betp@Gel), achieving multidimensional synergistic therapy through sustained drug release, local microenvironment modulation, and pathological pain alleviation.

XRD and FTIR results indicate Betp@Gel forms via stable coordination bonds between phosphate groups and Ca^2+^. Previous studies loaded protein drugs using Betp and CaCl_2_ coordination [37,38], but there have been no reports on the loading of hydrophobic drugs. TEM results of Betp@Gel and Cur/Betp@Gel revealed that both exhibit highly ordered nanofiber network structures. This three-dimensional porous network significantly enhanced drug-loading capacity. Rheological analysis further demonstrated that the storage modulus (G′) of both hydrogels was markedly higher than the loss modulus (G″), confirming their predominantly elastic mechanical behavior. Importantly, Cur loading did not significantly alter the rheological properties of the hydrogel, suggesting that Cur molecules may be embedded into the nanofiber network through non-covalent interactions (e.g., hydrophobic interactions or π-π stacking) rather than interfering with the gel self-assembly process. This characteristic is critical for maintaining the structural integrity of the drug-loaded system and provides a universal strategy for loading other hydrophobic drugs.

Regarding drug delivery performance, both in vitro and in vivo experiments revealed the sustained-release advantages of Betp@Gel. In vitro release experiments have shown that Cur/Betp@Gel can release Betp and Cur in vitro for more than 16 days. Cy7.5 fluorescent tracing showed that free Cy7.5 was metabolically cleared within 3 days in vivo, while the Cy7.5/Betp@Gel group maintained detectable signals for at least 5 days. Specifically, this supramolecular nanofiber hydrogel formed by non-covalent bond self-assembly delays drug release, while gradual hydrogel degradation enabled sustained drug release. The prolonged in vivo retention of Cy7.5/Betp@Gel underscores its potential to extend therapeutic exposure windows while reducing dosing frequency—a critical advantage for chronic conditions requiring long-term management. These hydrogels can be engineered to delay the release of drugs, ensuring a sustained therapeutic effect while minimizing potential side effects. One of the key advantages of supramolecular hydrogels is their ability to degrade in a controlled manner, which is crucial for avoiding long-term accumulation in tissues and reducing the risk of immunogenic reactions [39].

Cur/Betp@Gel's therapeutic effect stems from synergistic antioxidant (Cur) and anti-inflammatory (Betp) actions. Peripherally, ELISA and immunofluorescence showed Cur/Betp@Gel significantly reduced sciatic nerve pro-inflammatory cytokines (TNF-α, IL-1β, IL-6). This anti-inflammatory effect originates from Betp, the supramolecular hydrogel's gel-forming steroid component. While peripheral nerves possess some regenerative capacity, their repair critically depends on suppressing post-injury inflammation [10]. Immunofluorescence also revealed reduced iNOS in injured nerves, indicating sustained Cur release effectively exerts antioxidant effects in vivo. Although Cur's antioxidant properties were prominent in vitro, its in vivo efficacy may be constrained by the blood-nerve barrier. Betp@Gel's localized sustained-release compensates by maintaining effective Cur concentrations at the injury site, enabling continuous oxidative stress neutralization. In the critical acute phase following nerve injury, M2 macrophage polarization is key to scavenging debris and promoting regeneration. Using Arg-1 as an early marker, we demonstrate that Cur/Betp@Gel potently induces this pro-regenerative M2 phenotype, thereby creating a favorable foundation for nerve repair. Improved gastrocnemius muscle and sciatic nerve histology indirectly indicate that Cur/Betp@Gel may delay muscle atrophy and promote nerve regeneration through anti-inflammatory and antioxidant mechanisms. This "carrier-drug" functional complementarity offers a novel PNI treatment strategy, alleviating denervation-induced muscle atrophy [15,40].

There exists a significant correlation among the early excessive inflammatory response, the regenerative process, and the experience of pain [41]. In the initial stages of peripheral nerve damage during the acute phase of approximately seven days, involves an allergic response to mechanical and thermal stimuli. In the second stage, characterized by glutamatergic transmission, pain sensitivity generally returns to baseline levels within three to seven weeks. The third stage involves the progression to chronic pain [42]. Excessive inflammatory responses following the early inhibition of PNI can facilitate nerve healing and mitigate the incidence of chronic neuropathic pain subsequent to peripheral nerve injury [11,43,44]. NP occurs after nerve injury, and harmful changes occur in the injured neurons, which then affect the downward regulatory pathways of the body's sensory system and the central nervous system [45,46]. PNI triggers a long-term pro-inflammatory response of spinal glial cells, which promotes chronic pain [47]. Effective treatment to suppress inflammation can largely repair injured nerves and reduce the occurrence of chronic rational pain after PNI. At the central level, reduced GFAP/Iba-1-positive areas in the spinal dorsal horn indicate that Cur/Betp@Gel suppresses astrocyte and microglial hyperactivation, thereby blocking spinal sensitization in neuropathic pain. This "peripheral-central" dual regulatory mechanism overcomes the limitations of traditional single-target therapies.

The core innovation of this study is to form supramolecular hydrogels with Betp and Cacl2, which are commonly used in clinical practice, and then loading natural drugs Cur to build an injectable, long-acting and multi-functional treatment system. Compared to systemic administration, localized Cur/Betp@Gel application avoids hepatic first-pass effects and reduces systemic toxicity. Relative to single-component therapies (e.g., Cur alone or plain hydrogel), its synergistic anti-inflammatory, antioxidant, and pain-alleviating effects significantly enhance therapeutic outcomes (e.g., improved SFI index and pain thresholds). Blood biochemistry and histopathology confirmed its biosafety, laying a foundation for clinical translation.

The primary limitation of this study is the exclusive use of male rats. Although this approach minimizes variability associated with the female estrous cycle, it fails to account for sex-based differences in pain perception and nerve repair mechanisms. To thoroughly evaluate the treatment's applicability, future research should incorporate both male and female subjects.

## 5 Conclusion

In this study, we developed an innovative drug-delivery hydrogel (Cur/Betp@Gel) by exploiting the molecular coordination bond between the clinical corticosteroid Betp and CaCl_2_. This unique "therapeutic depot" not only provides a biocompatible and clinically translatable platform but also enables the sustained co-delivery of Betp and curcumin over at least five days in vivo. The synergistic action of the anti-inflammatory Betp and the antioxidant curcumin effectively mitigated local oxidative stress and inflammation, protected neural structures, prevented muscle atrophy, and inhibited neuroinflammation and central sensitization. Consequently, this combination significantly enhanced peripheral nerve repair and alleviated chronic neuropathic pain. Crucially, the long-lasting release profile of our hydrogel addresses a key clinical challenge by minimizing the need for repeated injections, thereby reducing the risk of iatrogenic nerve injury. Our findings underscore the substantial promise of this Betp-based hydrogel as a transformative strategy for treating peripheral nerve injuries and pathological neuralgia.

## CRediT authorship contribution statement

**Anqi Liu:** Investigation, Funding acquisition, Formal analysis, Data curation, Conceptualization. **Kui Sheng:** Formal analysis, Data curation, Conceptualization. **Huihui Li:** Resources, Project administration, Methodology, Investigation. **XinYang Zhao:** Resources, Project administration, Methodology. **Xiaojun Zhang:** Project administration, Methodology, Investigation. **Haitao Su:** Formal analysis, Data curation, Conceptualization. **Tsring Samdrup:** Funding acquisition, Formal analysis, Data curation. **Ye Zhang:** Supervision, Software, Resources, Project administration. **Xianwen Hu:** Writing – review & editing, Writing – original draft, Visualization, Validation, Supervision. **LiJun Weng:** Writing – review & editing, Visualization, Validation, Supervision. **YaGuang Wang:** Writing – review & editing, Writing – original draft, Visualization, Validation, Supervision.

## Ethics approval and consent to participate

The animal use protocol in this paper has been reviewed and approved by the Animal Care and Use Committee of Anhui Medical University.

## Funding

The author(s) disclosed receipt of the following financial support for the research, authorship, and/or publication of this article: This work was supported by grants from the 10.13039/501100003995Natural Science Foundation of Anhui Province (grant no. 2308085MH238 and grant no. 2408085MH212), the 10.13039/501100018543Natural Science Foundation of Tibet Autonomous Region (grant no.XZ2024ZR-ZY059(Z)).

## Declaration of competing interest

The authors declare that they have no known competing financial interests or personal relationships that could have appeared to influence the work reported in this paper.

## Data Availability

Data will be made available on request.
