# Supplementary material for: Injectable anti-inflammatory, antioxidant supramolecular nanofiber hydrogel for peripheral nerve injury repair and neuropathic pain relief
Source: Mater Today Bio. 2026 Jan 7;37:102780. doi: 10.1016/j.mtbio.2026.102780 (PMC12856178; doi:10.1016/j.mtbio.2026.102780)
Supplement: Multimedia component 1 [file mmc1.pdf]

Supporting Information

Injectable anti-inflammatory, antioxidant supramolecular nanofiber hydrogel for peripheral nerve injury repair and neuropathic pain relief

Anqi Liu [a,b,c1], Kui Sheng [a,b1], Huihui Li [b,d1], XinYang Zhao [a,b,c], Xiaojun Zhang [a,b,c], Haitao Su [a,b,c], Tsring Samdrup [d], Ye Zhang [a,b,c], Xianwen Hu [a,b,c*], LiJun Weng [a,b,c*], YaGuang Wang [a,b,c*]

a. Department of Pain, The Second Affiliated Hospital of Anhui Medical University, 678 Furong Road, Hefei 230601, PR China
b. Department of Anesthesiology, The Second Affiliated Hospital of Anhui Medical University, 678 Furong Road, Hefei 230601, PR China
c. Key Laboratory of Anesthesiology and Perioperative Medicine of Anhui Higher Education Institutes, Anhui Medical University, 678 Furong Road, Hefei 230601, PR China
d. Department of Anesthesiology, Fuyang People's Hospital Affiliated to Anhui Medical University, 501 Sanqing Road, Fuyang, Anhui, China.
e. Department of Anesthesiology, People's Hospital of Shannan City, Tibet Autonomous Region

1 These authors contributed equally to this work and should considered co-first authors.
* Corresponding authors. E-mail: Xianwen Hu (huxianwen001@163.com), Lijun Weng (wenglijun@ahmu.edu.cn) Yaguang Wang (wangyaguang@ahmu.edu.cn)

This file includes:

1. Equipment
2. Materials and methods
3. Supporting Figures (**Figure S1-S9**)

1. Equipment

^{1}H NMR and ^{13}C NMR spectra were obtained on a Bruker AV 400. Rheology measurements were obtained on a MCR302 (Anton Paar). The circular dichroism (CD) spectra were obtained on Chirascan V100 (Applied Photophysics Ltd, UK).

2. Materials and methods

MS: calculated for compound NapFFY [(M+H+)]: 644.2716, [(M+Na+)]: 666.2582; obsvd. HR-MS [(M+H+)]: m/z 644.2764, [(M+Na+)]: m/z 666.2584. (Fig. S1)

^{1}H NMR of Bet-p (400 MHz, D2O) 1H NMR (400 MHz, D2O) δ 7.46 (d, J = 10.1 Hz, 1H), 6.34 (dd, J = 10.1, 2.0 Hz, 1H), 6.14 (t, J = 1.7 Hz, 1H), 4.74 (d, J = 5.1 Hz, 1H), 4.54 (dd, J = 19.1, 4.7 Hz, 1H), 4.31 (ddd, J = 9.9, 4.2, 2.2 Hz, 1H), 2.64 (td, J = 13.4, 5.9 Hz, 1H), 2.57 – 2.43 (m, 1H), 2.43 – 2.35 (m, 1H), 2.11 (h, J = 7.5 Hz, 1H), 1.96 (ddt, J = 28.9, 18.6, 8.2 Hz, 4H), 1.52 (d, J = 13.3 Hz, 1H), 1.47 (s, 3H), 1.42 (dt, J = 12.8, 6.3 Hz, 1H), 1.14 (t, J = 10.5 Hz, 1H), 1.05 (d, J = 7.4 Hz, 3H), 1.00 (s, 3H).

^{13}C NMR of Bet-p (100 MHz, D2O) 13C NMR (100 MHz, D2O) δ 211.8, 211.7, 188.9, 171.6, 156.2, 128.5, 123.7, 103.1, 101.4, 89.4, 71.6, 71.2, 70.0, 70.0, 49.1, 48.9, 48.3, 47.6, 43.4, 36.1, 34.6, 33.6, 33.4, 31.1, 27.8, 22.4, 22.4, 19.1, 17.0.

3. Supporting Figures

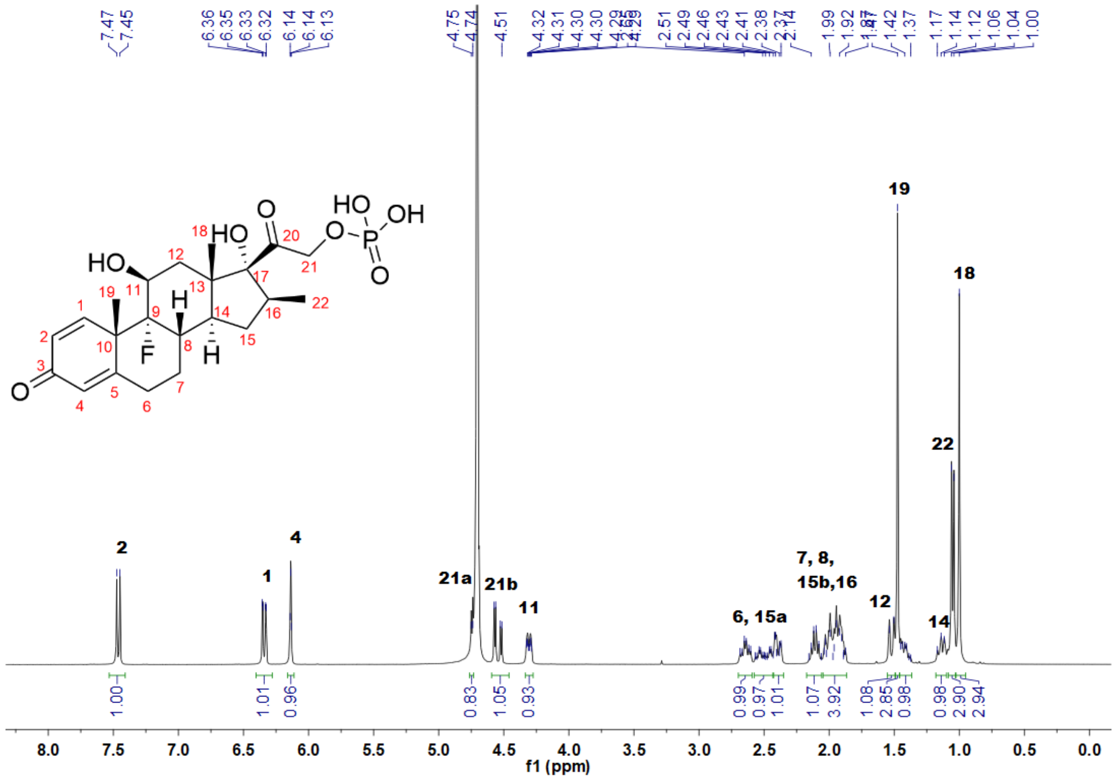

Figure S1. ^{1}H NMR spectrum of Betp in D*2O*.

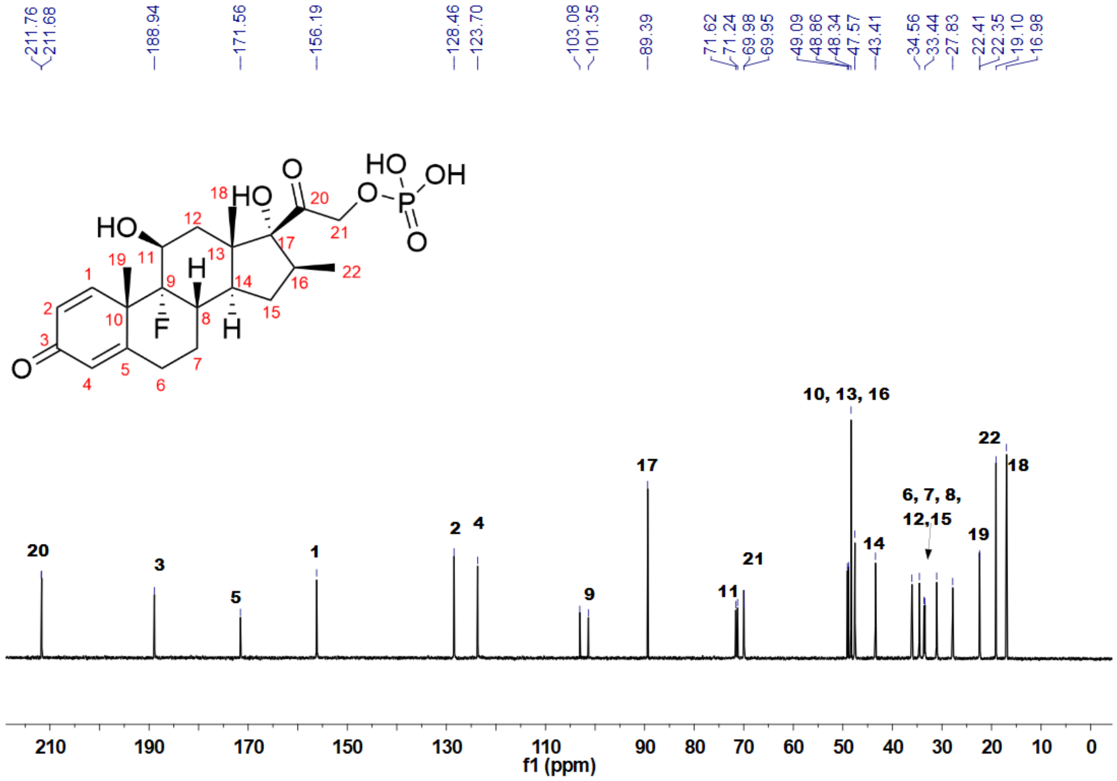

Figure S2. ^{1}C NMR spectrum of Betp in D*2O*.

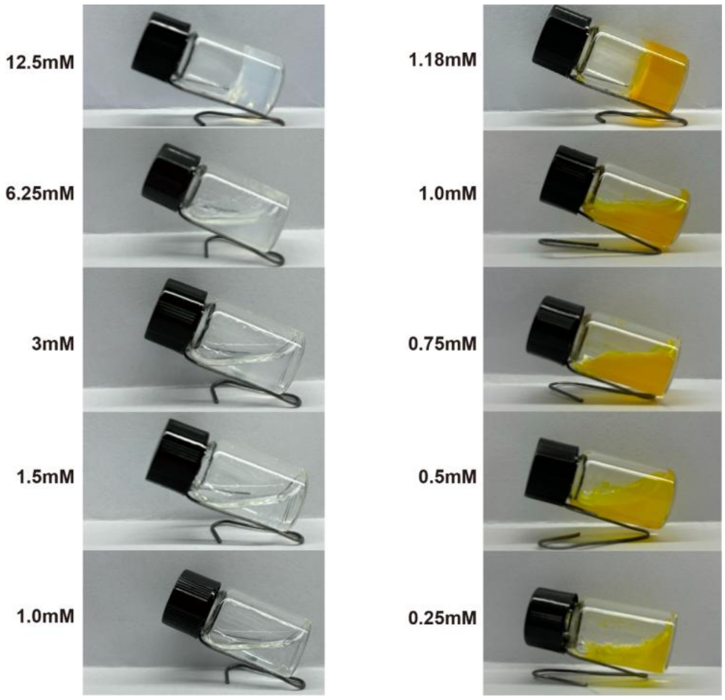

Figure S3. Photographs showing the Betp@Gel and Cur/Betp@Gel with different concentrations of Betp and Cur.

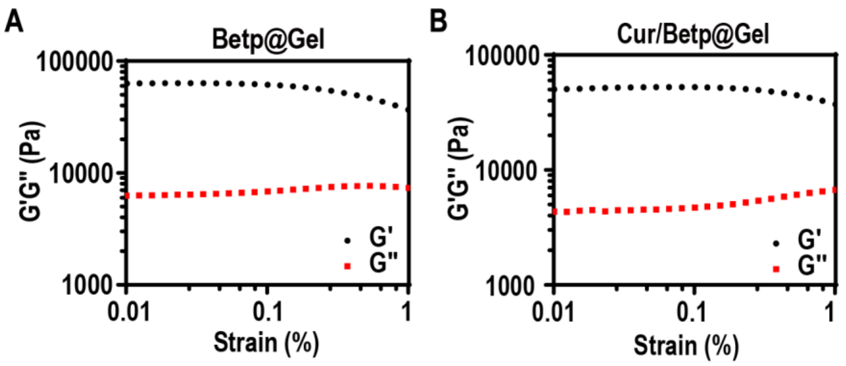

Figure S4. Storage modulus (G’) and loss modulus (G’’) of Betp@Gel. (A) and Cur/Betp@Gel (B) at different strain rates with a fixed frequency of 1.0 Hz.

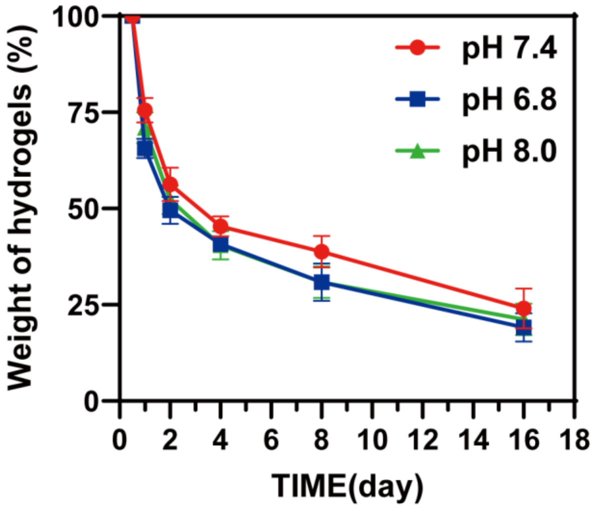

Figure S5. In vitro degradation kinetics of Cur/Betp@Gel in phosphate-buffered saline (PBS) under varying pH conditions.

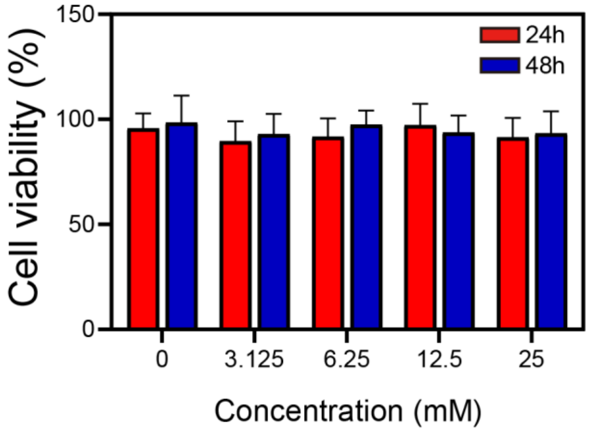

Figure S6. Effect of different concentrations of compound Betp@Gel or equivalent concentrations of Betp and $CaCl_2$ mixtures for 24 h on F11 cells viability. The F11 cell viability was determined by CCK8 assay. Data were expressed as the mean ± SEM of 6 independent experiments.

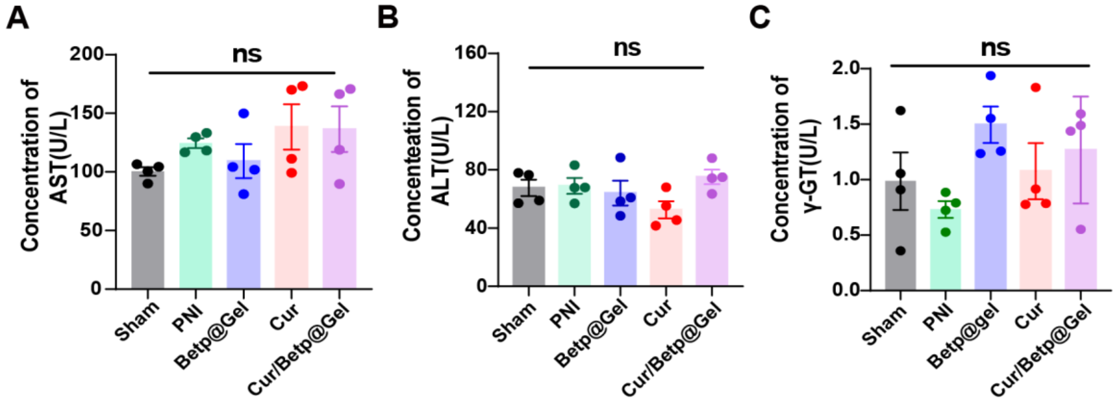

Figure S7. Levels of ASL, ALT, and γ-GT in rat serum 5 days post-surgery across groups.

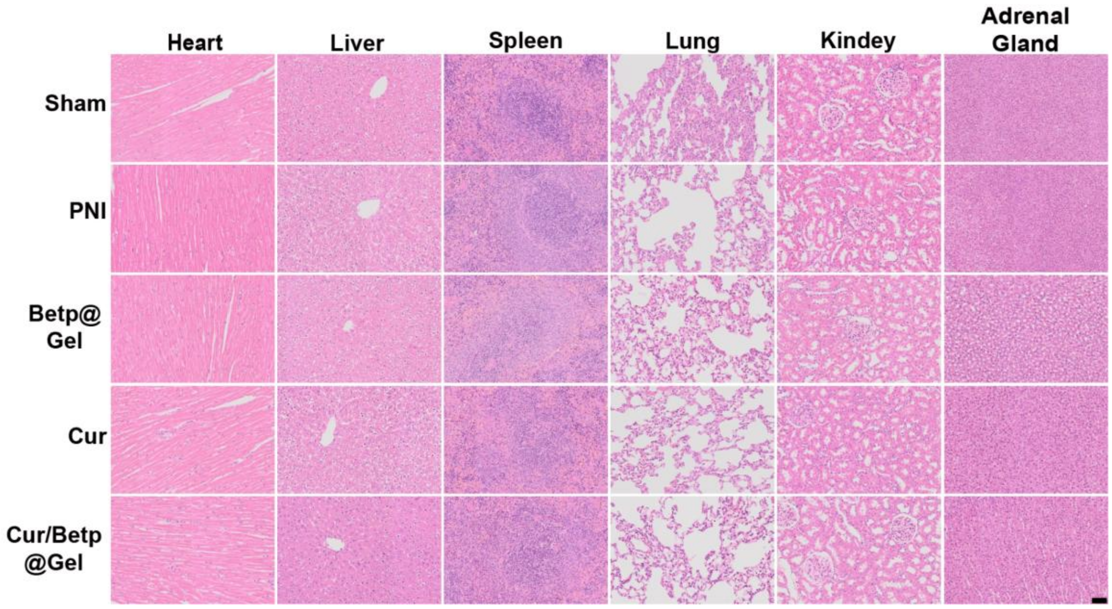

Figure S8. H&E stained images of major organs (heart, liver, spleen, lung, kidney,adrenal gland) harvested from five groups of rats sacrificed 28 days post-PNI. Scale bar: 50 μm.

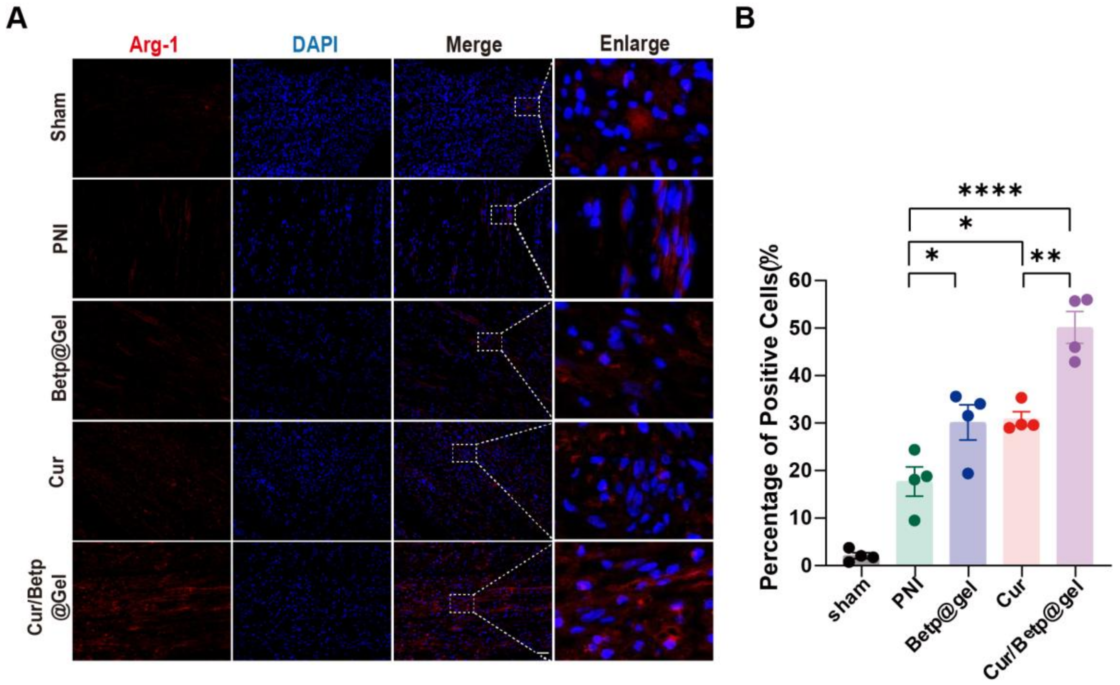

Figure S9. Cur/Betp@Gel can promote the M2 polarization of macrophages in injured nerves. (A) Representative immunofluorescence staining image of Arg-1 in the injured sciatic nerve at 7 days post-PNI. Scale: 50 μm. (B) Statistical analysis of the Arg-1 positive area (n = 4). Data are presented as the means ± SEM. $^{*}P < 0.05$, $^{**}P < 0.01$, $^{***}P < 0.001$, $^{****}P < 0.0001$.
